# Theiler's Virus-Mediated Immunopathology in the CNS and Heart: Roles of Organ-Specific Cytokine and Lymphatic Responses

**DOI:** 10.3389/fimmu.2018.02870

**Published:** 2018-12-10

**Authors:** Seiichi Omura, Eiichiro Kawai, Fumitaka Sato, Nicholas E. Martinez, Alireza Minagar, Mahmoud Al-Kofahi, J. Winny Yun, Urska Cvek, Marjan Trutschl, J. Steven Alexander, Ikuo Tsunoda

**Affiliations:** ^1^Department of Microbiology, Kindai University Faculty of Medicine, Osaka, Japan; ^2^Department of Microbiology and Immunology, Center for Molecular and Tumor Virology, Center for Cardiovascular Diseases and Sciences, Louisiana State University Health Sciences Center-Shreveport, Shreveport, LA, United States; ^3^Department of Neurology, Louisiana State University Health Sciences Center-Shreveport, Shreveport, LA, United States; ^4^Department of Molecular and Cellular Physiology, Louisiana State University Health Sciences Center-Shreveport, Shreveport, LA, United States; ^5^Department of Computer Science, Louisiana State University Shreveport, Shreveport, LA, United States

**Keywords:** adhesion molecules, animal models, blood-brain barrier, computational analysis, GLYCAM1, LYVE1, *Picornaviridae* infection, unsupervised analysis

## Abstract

Theiler's murine encephalomyelitis virus (TMEV) induces different diseases in the central nervous system (CNS) and heart, depending on the mouse strains and time course, with cytokines playing key roles for viral clearance and immune-mediated pathology (immunopathology). In SJL/J mice, TMEV infection causes chronic TMEV-induced demyelinating disease (TMEV-IDD) in the spinal cord about 1 month post-inoculation (p.i.). Unlike other immunopathology models, both pro- and anti-inflammatory cytokines can play dual roles in TMEV-IDD. Pro-inflammatory cytokines play beneficial roles in viral clearance while they are also detrimental in immune-mediated demyelination. Anti-inflammatory cytokines suppress not only protective anti-viral immune responses but also detrimental autoreactive immune responses. Conversely, in C3H mice, TMEV infection induces a non-CNS disease, myocarditis, with three distinctive phases: phase I, viral pathology with interferon and chemokine responses; phase II, immunopathology mediated by acquired immune responses; and phase III, cardiac fibrosis. Although the exact mechanism(s) by which a single virus, TMEV, induces these different diseases in different organs is unclear, our bioinformatics approaches, especially principal component analysis (PCA) of transcriptome data, allow us to identify the key factors contributing to organ-specific immunopathology. The PCA demonstrated that *in vitro* infection of a cardiomyocyte cell line reproduced the transcriptome profile of phase I in TMEV-induced myocarditis; distinct interferon/chemokine-related responses were induced *in vitro* in TMEV-infected cardiomyocytes, but not in infected neuronal cells. In addition, the PCA of the *in vivo* CNS transcriptome data showed that decreased lymphatic marker expressions were weakly associated with inflammation in TMEV infection. Here, dysfunction of lymphatic vessels is shown to potentially contribute to immunopathology by delaying the clearance of cytokines and immune cells from the inflammatory site, although this can also confine the virus at these sites, preventing virus spread via lymphatic vessels. On the other hand, in the heart, dysfunction of lymphatics was associated with reduced lymphatic muscle contractility provoked by pro-inflammatory cytokines. Therefore, TMEV infection may induce different patterns of cytokine expressions as well as lymphatic vessel dysfunction by rather different mechanisms between the CNS and heart, which might explain observed patterns of organ-specific immunopathology.

## Introduction

### Theiler's Murine Encephalomyelitis Virus (TMEV) Induces Distinct Organ-Specific Diseases

**Figure 1:**
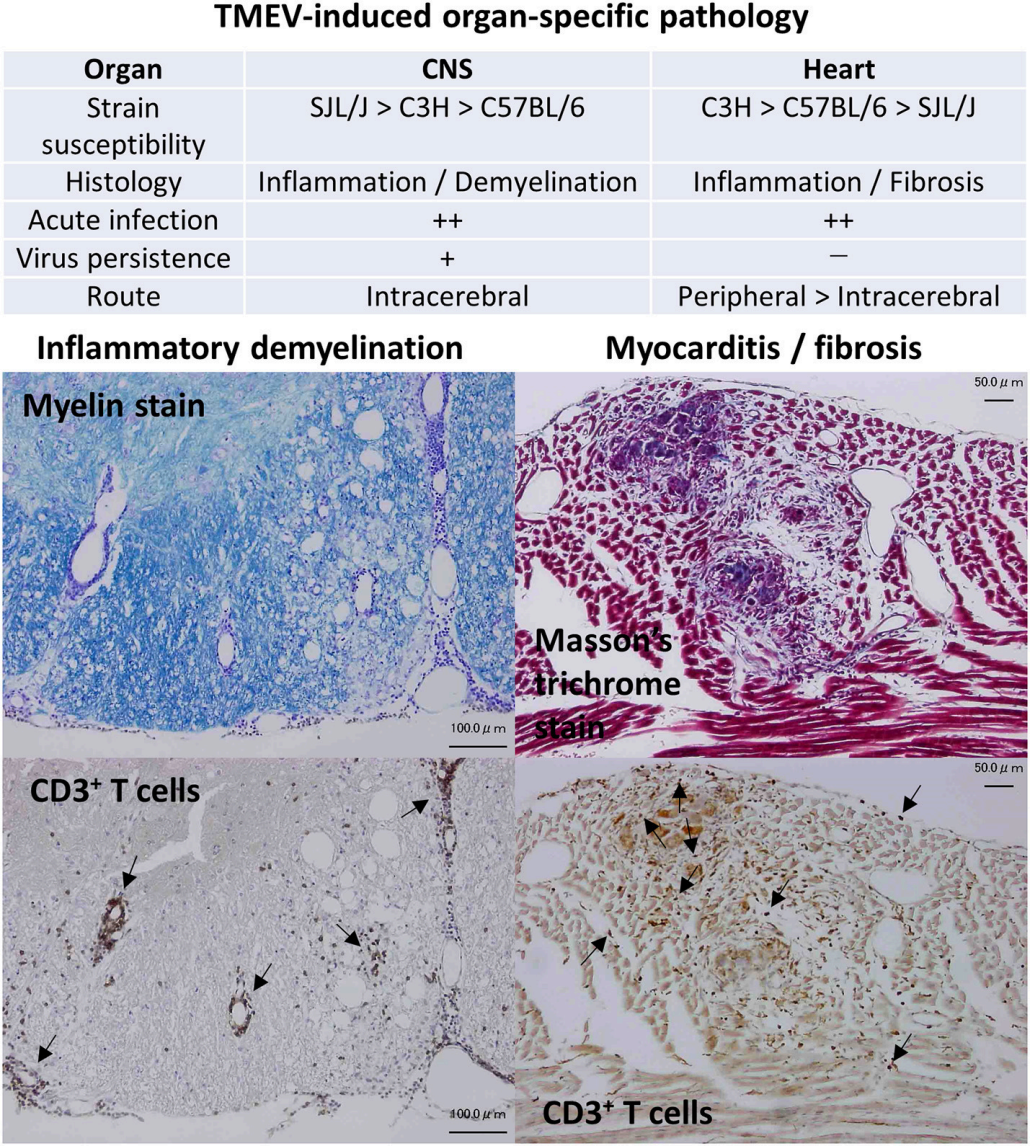
Organ-specific pathology induced by Theiler's murine encephalomyelitis virus (TMEV). TMEV induces pathology in two organs: inflammatory demyelination in the central nervous system (CNS) and inflammation followed with fibrosis in the heart, whose susceptibilities differ among mouse strains (9, 10). Although TMEV can infect the CNS and the heart during the acute phase, persistent viral infection is observed only in the CNS. CNS disease can be induced only by intracerebral inoculation. On the other hand, both peripheral and intracerebral routes of viral inoculation result in myocarditis, while peripheral inoculation induces more severe cardiac disease. (**Left**) Inflammatory demyelination in the spinal cord of TMEV-induced demyelinating disease (TMEV-IDD). Luxol fast blue stain. CD3 immunohistochemical staining of consecutive sections showed that T cells were present in perivascular cuffing and meningitis (Arrows). Bar: 100 μm (**Right**) Inflammation and fibrosis in the heart during phase III of TMEV-induced myocarditis. Masson's trichrome stain. CD3 immunohistochemical staining showed T cell infiltration (Arrows) in the heart. Bar: 50 μm.

Theiler's murine encephalomyelitis virus (TMEV) is a non-enveloped, single-stranded positive-sense RNA virus that belongs to the order *Picornavirales*, family *Picornaviridae*, genus *Cardiovirus*. Historically, Max Theiler discovered the Theiler's original (TO) strain of TMEV as an agent that induces acute polioencephalomyelitis in the central nervous system (CNS) of mice in 1934 (1–3). Since TMEV infects the gastrointestinal tract and induces an acute CNS disease similar to poliovirus (family *Picornaviridae*, genus *Enterovirus*), TMEV was originally classified into the genus *Enterovirus* and used as an animal model for poliomyelitis. In 1952, Joan Daniels reported that the Daniels (DA) strain of TMEV causes myositis in the skeletal muscle and a chronic inflammatory demyelinating disease in the spinal cord (4), the latter of which has been called TMEV-induced demyelinating disease (TMEV-IDD) and used as a viral model for multiple sclerosis (MS) (5–7), first by Howard Lipton in 1972. In 1996, Gómez et al. demonstrated that TMEV causes inflammation not only in the skeletal muscle (i.e., myositis) but also in the heart muscle (i.e., myocarditis) (8). Since 2014, TMEV-induced myocarditis has been applied as a viral model for myocarditis (9) (Figure 1). The resistance/susceptibility to TMEV-induced organ-specific pathology has been known to differ among mouse strains. The resistance to persistent CNS infection maps genetically to major histocompatibility complex (MHC) class I, *H-2D* region (3). The *H-2* background also appears to influence myositis and myocarditis, although studies using congenic mice are necessary to determine the precise role of MHC molecules (8).

In general, viruses infect limited species and induce diseases in an isolated group of organs. The determination of the mechanism(s) of such organ-specific tropism/pathogenesis of virus infections could powerfully inform the development of treatments and methods of prevention for viral infections: currently the precise mechanisms of many types of viral pathogenesis still remain unknown. TMEV is a natural enteric pathogen of mice (11) and has been isolated from trapped wild mice (12), while no TMEV-induced disease has been reported in the wild. TMEV has been shown to infect only mice, and not other species *in vivo* (with a few exceptions) and causes distinct maladies that mimic human diseases (3). In experimental mice, intracerebral inoculation of TMEV results in CNS viral infection as well as viremia and induces diseases in the CNS and the heart (13). On the other hand, peripheral inoculation, such as intraperitoneal or intravenous injection, causes myocarditis more efficiently (9), but rarely causes CNS infection. Thus, TMEV has high neurotropism and high neurovirulence, but low neuroinvasiveness, despite the fact that TMEV can use at least three routes to gain access to the CNS: neural spread, hematogenous spread, and olfactory route (14). Low neuroinvasiveness of peripherally inoculated TMEV can be explained by the fact that vascular endothelial cells are not permissive for TMEV infection *in vivo* (15). Here, although TMEV can still invade the CNS hematogenously, using infected macrophages as Trojan horse (3), this is not an efficient way to achieve fast and successful viral invasion into the CNS. TMEV infects only certain cell types in restricted organs *in vivo*, although TMEV can infect most cell lines derived from various organs and different host species, even insect cells (with the exception of T cells *in vitro*) (15).

### TMEV-Induced CNS Disease

TMEV is divided into two subgroups: the TO and GDVII, based on its neurovirulence following intracerebral inoculation. The GDVII subgroup, including GDVII and FA strains, causes acute fatal polioencephalomyelitis and kills all mice following intracerebral infection. One plaque forming unit (PFU) of GDVII virus is enough to kill mice by induction of neuronal apoptosis and axonal injury without inducing acquired immune responses (16). The TO subgroup, including DA and BeAn strains, induces a biphasic disease in susceptible mouse strains (highly susceptible, SJL/J mice; and intermediate susceptible, C3H mice), following intracerebral injection (17). During the acute phase, about 1 week post-inoculation (p.i.), TMEV infects neurons and induces neuronal apoptosis, neuronophagia, and inflammation, mainly in the gray matter of the brain, including the hippocampus and cerebral cortex (polioencephalitis), while induction of TMEV-specific cellular and humoral immune responses is accompanied by the clearance of the virus from the brain. Thereafter, a low level of TMEV can persistently infect oligodendrocytes and microglia/macrophages in the white matter of the spinal cord of susceptible mice, and recruit anti-viral immune cells into the infected regions, particularly ventrolateral funiculus of the thoracic segments, leading to inflammatory demyelination during the chronic phase, about 1 month p.i. (3).

During the acute phase of TMEV infection, CD4^+^ and CD8^+^ T cells and anti-viral antibodies enter the CNS, contributing to viral clearance from the gray matter without causing overt immune-mediated tissue damage (immunopathology). During the chronic phase, however, these same immune effector components are detected in the white matter, and play key roles in immunopathology (18). Overall, gain-of-function and loss-of-function approaches to clarify the roles of immune effector cells, antibodies, and cytokines, have demonstrated that anti-viral pro-inflammatory effector molecules/cells, including CD4^+^ T helper (Th)1, cells and CD8^+^ cytotoxic T lymphocytes (CTLs), and antibodies play protective roles during the acute phase (7). For example, in GDVII virus infection, lack of CNS infiltrating immune cells results in acute fatal polioencephalitis (19), which has been associated with altered mRNA expression levels of cytokines, but not chemokines (20), as well as induction of transforming growth factor (TGF)-β1 protein in the neurons (21). On the other hand, during the chronic phase, these immune effector molecules/cells could play detrimental roles causing immunopathology in a bystander fashion and/or determinant (epitope) spreading to myelin antigens (22, 23), although the precise mechanisms of immunopathology remain unknown. While these immune effector molecules/cells (Th1, CTL, and antibody) seem to play both protective anti-viral and detrimental immunopathogenic roles in TMEV-IDD, anti-inflammatory cells including regulatory T cells (Tregs) can also be beneficial and detrimental depending on the disease phases in TMEV infection (24).

### TMEV-Induced Myocarditis

Myocarditis is an inflammatory disease in the heart caused by microbial infections or autoimmunity and affects about 2 million people in the United States (25, 26). Among viruses, the picornavirus family, especially coxsackievirus B, is a well-known pathogen of myocarditis (27). In general, regardless of viral families or species, viral myocarditis has been proposed to be divided into three phases (28). In phase I (around 4 days p.i., experimentally), the virus infects and replicates in the heart, damaging cardiomyocytes, while innate immune responses against the virus are induced. In phase II, anti-viral T-cell and antibody responses are induced (after 5 or more days p.i.), with the penetration of these effector components into the heart. Under pathologic conditions, anti-viral immune responses not only clear the virus but also damage infected and uninfected cardiomyocytes by anti-viral CTLs and in a bystander fashion, respectively. This can be followed by induction of autoimmune responses to the heart reflecting determinant spreading and/or molecular mimicry between the virus and heart antigens. When the tissue damage caused in phase I and/or II is severe, cardiac remodeling and fibrosis with or without low-grade viral persistence occur, which can lead to dilated cardiomyopathy (phase III). Ideally, each patient with myocarditis should be treated depending on the phase (29): phase I, antiviral; phase II, immunomodulation; and phase III, standard heart failure therapy (e.g., immunosuppression may be appropriate for phase II, but will enhance virus replication in phase I). However, finding effective therapies has remained challenging because the phase-specific biomarkers and pathogenesis of myocarditis have not been conclusively identified (30), while serum cardiac troponin and creatine kinase, electrocardiogram and echocardiography, and the endomyocardial biopsy have been helpful, to some extent, for diagnosing myocarditis (31, 32).

To clarify the pathogenesis and discover the phase-specific biomarkers, we established a murine model for viral myocarditis using TMEV (9, 28, 33), which has unique characteristics not seen in most other animal models. For example, (1) most animal models don't have three phases; (2) fail to reproduce clinical and immunological findings in human viral myocarditis; and (3) fail to use a “natural” pathogen of the host, thus the TMEV model is possibly more relevant to human natural infections. Generally, peripheral injection (e.g., intraperitoneal) of TMEV in mice can efficiently cause inflammation in the heart, but not in the CNS (8, 13), while intracerebral injection of TMEV also causes myocarditis due to acute viremia. Susceptibilities to TMEV-induced myocarditis differ among mouse strains: the highly susceptible C3H strain, the intermediate susceptible C57BL/6 strain, and the highly resistant SJL/J strain. C3H mice develop all three phases, while SJL/J mice develop only phase I and C57BL/6 mice develop phases I and II; the different genetic susceptibilities to viral myocarditis has also been demonstrated in humans (34). TMEV-induced myocarditis can be divided into three phases as in human myocarditis. In phase I, innate immune molecules [interferon (IFN)-induced genes [e.g., interferon regulatory factor 7 (*Irf7*), interferon-induced protein with tetratricopeptide repeats 1 (*Ifit1*), and *Ifit3*] and chemokine genes [e.g., chemokine (C-X-C motif) ligand 9 (*Cxcl9*), *Cxcl10*, and chemokine (C-C motif) ligand 5 (*Ccl5*)] that can recruit Th1 and natural killer T (NKT) cells] were upregulated prior to immune cell infiltration in the heart. In phase II, T-cell infiltrates were observed with upregulation of pro-inflammatory IFN-γ pathway genes, followed by upregulation of cardiac remodeling genes (e.g., *Mmp12* and *Gpnmb*) in phase III. Among transgenic and knockout (KO) mice infected with TMEV, NKT KO mice developed more severe myocarditis with lower ejection fraction in echocardiography than wild-type mice (10).

### Lymphatics and Viral Infections

The afferent lymphatic vessels transport interstitial fluid and antigens from tissues to lymph nodes and have specialized capillaries with an open structure; antigen transport to the draining lymph nodes is required to generate antigen-specific immune responses (35). Cancer cells and pathogens often “hijack” this transport system to achieve systemic spread (36), while dissemination to the blood circulation is first blocked at regional lymph nodes. In viral infections, while the mechanisms that limit systemic viral spread have not been studied extensively, several mechanisms have been proposed recently. Kastenmüller et al. (37) showed that vaccinia virus injected subcutaneously in mice was acquired by CD169^+^ subcapsular sinus macrophages in the regional lymph nodes, but not in the spleen, 4 hours (h) p.i. Since local depletion of macrophages by clodronate-loaded liposomes resulted in viral spreading to the spleen, these results suggest that systemic viral spread ensues in the absence of effective viral capture by macrophages. On the other hand, Loo et al. (38) demonstrated that vaccinia virus infection by scarification, which did not spread the virus to draining lymph nodes, induced remodeling of the pre-existing cutaneous lymphatic vasculature, but not lymphangiogenesis. The remodeling was coincident with a rapid reduction in fluid transport, suggesting that lymphatic vessels negatively modulate fluid transport following viral infection in the skin, to limit the spread of viral particles into lymph nodes. Lymphatic vessel remodeling can result in not only compartmentalization of infectious virus, but also an accumulation of inflammatory mediators in the skin, which affect anti-viral immunity and immunopathology.

In the following sections, we will introduce our bioinformatics analyses of both supervised (such as heat map and *k*-means clustering) and unsupervised [particularly principal component analysis (PCA)] approaches to identify factors that contribute to organ-specific viral pathology. Previously, using these computational analyses, we were able to identify and rank key molecules involved in MS (39), stroke (40), and myocarditis (33). Here, we focus on two potential candidate factors contributing to organ-specific viral pathology: (1) innate immune responses by the major cell type of each organ, i.e., cardiomyocyte in the heart vs. neuron in the CNS; and (2) lymphatic vessel dysfunction induced by cytokines in the heart vs. downregulation of neuro-lymphatic molecules in the CNS.

## Cell-type Specific Innate Immune Responses in TMEV Infection

### TMEV Infects and Damages Cardiomyocytes *in vitro*

**Figure 2:**
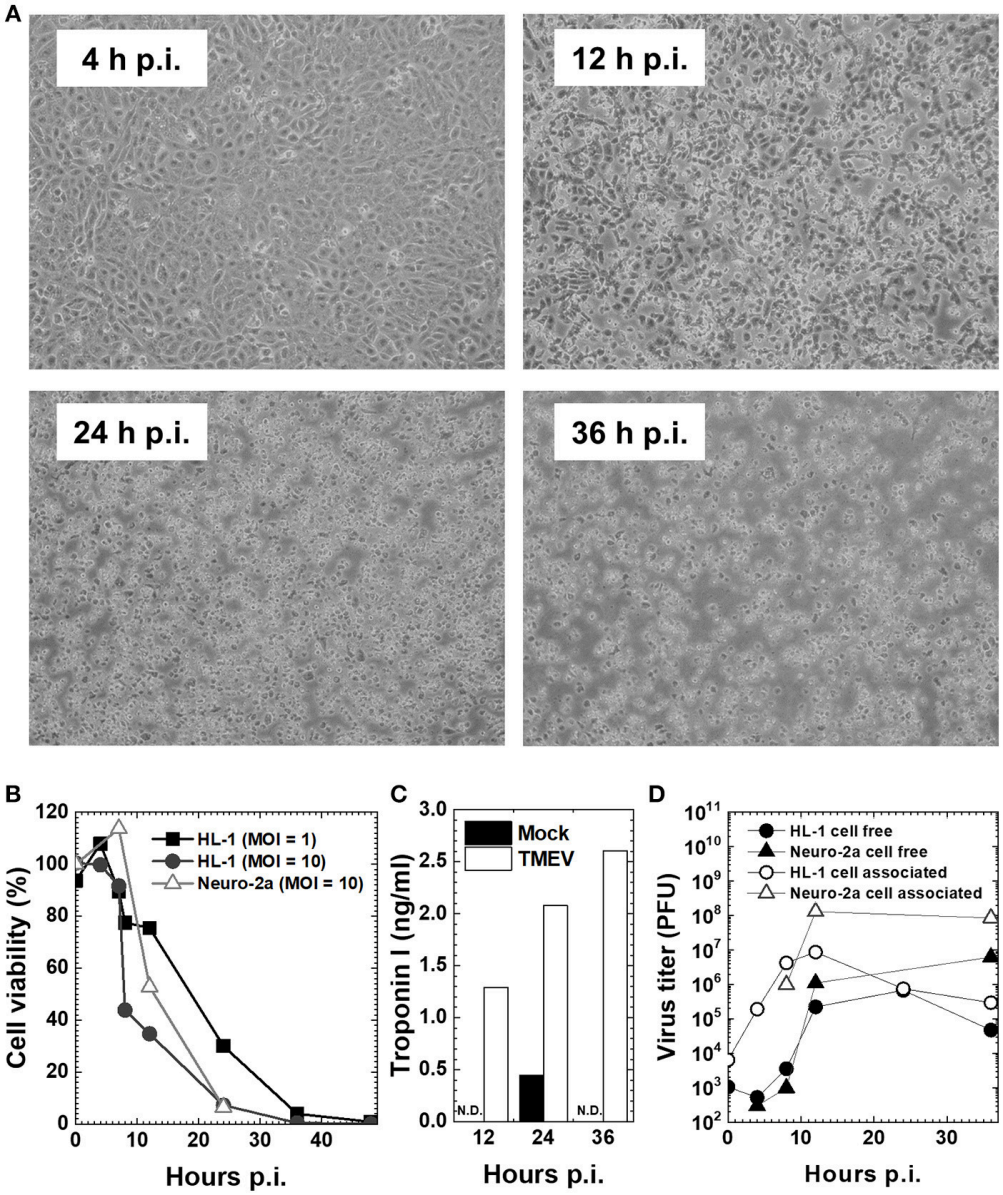
Cardiomyocyte cell line HL-1 infection with the Daniels (DA) strain of TMEV (33, 42). **(A)** Confluent HL-1 cell monolayer infected with TMEV at a multiplicity of infection (MOI) = 10 showed no changes at 4 hours (h) post-inoculation (p.i.). Cytopathic effect (CPE), including the rounding up and detachment of cells from the culture dish, was observed at 12 h p.i., which developed cell lysis in most cells at 36 h p.i. **(B)** HL-1 cells and neuroblastoma cell line Neuro-2a were infected with TMEV at an MOI = 1 or 10. Cell viability was determined with trypan blue dye exclusion assays. Cell viability of both HL-1 and Neuro-2a cells decreased at 12 h p.i. and most cells died at 36 h p.i. **(C)** The concentration of cardiac troponin I in cell culture supernatants determined by an enzyme-linked immunosorbent assay (ELISA) was detectable in TMEV-infected HL-1 cells (open column), but not detectable (N.D.) in mock-infected HL-1 cells (closed column) or infected Neuro-2a cell culture (data not shown). **(D)** Viral titers of cell-free (•, ▴) and cell-associated virus (°, Δ) in HL-1 or Neuro-2a cell culture were determined by plaque assays with baby hamster kidney (BHK)-21 cells (24). In both HL-1 and Neuro-2a cells, cell-free virus titers increased substantially at 12 h p.i., while cell-associated viral titers increased at 8 h p.i. and peaked at 12 h p.i.

The TMEV-induced myocarditis model *in vivo* is complemented by the *in vitro* model using a mouse cardiomyocyte cell line, HL-1, which was established by Dr. William C. Claycomb (Louisiana State University Health Sciences Center, New Orleans, LA) from an AT-1 subcutaneous tumor of a C57BL/6J mouse. HL-1 cells retain a differentiated cardiomyocyte phenotype and show contractile activity *in vitro* (41). To see the effects (innate immune responses and viral pathology) of direct virus infection without the involvement of immune cells (phase I mimic), we infected HL-1 cells, at a multiplicity of infection (MOI) = 1 or 10. TMEV infection induced cytopathic effects (CPE) on HL-1 cells, which became obvious 12 h p.i. (Figure 2A), while the cell viability started to decrease 8 h p.i., with most cells dying 36 h p.i. (Figure 2B). CPE was accompanied by the detection of cardiac troponin in the culture supernatants of HL-1 cells, which was measured by an enzyme-linked immunosorbent assay (ELISA) using the Ultra Sensitive Mouse Cardiac Troponin-I ELISA Kit (Life Diagnostics, West Chester, PA) (Figure 2C) (33). We also determined virus replication by plaque assays, using supernatants for cell-free virus and cell lysates for cell-associated virus (Figure 2D). Cell-free virus titers increased substantially 12 h p.i., which reflected a loss of plasma cell membrane integrity and showed similar kinetics with supernatant troponin concentrations. Cell-associated viral titers increased 8 h p.i. and peaked 12 h p.i., which was associated with the cell viability. In these assays, we also used a murine neuroblastoma cell line, Neuro-2a (43), since TMEV is known to infect neurons *in vitro* as well as during the acute phase following intracerebral infection *in vivo*. TMEV-infected Neuro-2a cells had similar kinetics of cell viability and viral replication to those of HL-1 cells, while cardiac troponin was not detectable in Neuro-2a cells regardless of infection, as expected (Figures 2B–D).

### Innate Immunity-Related Genes Are Upregulated Only in Cardiomyocytes Infected With TMEV

**Figure 3:**
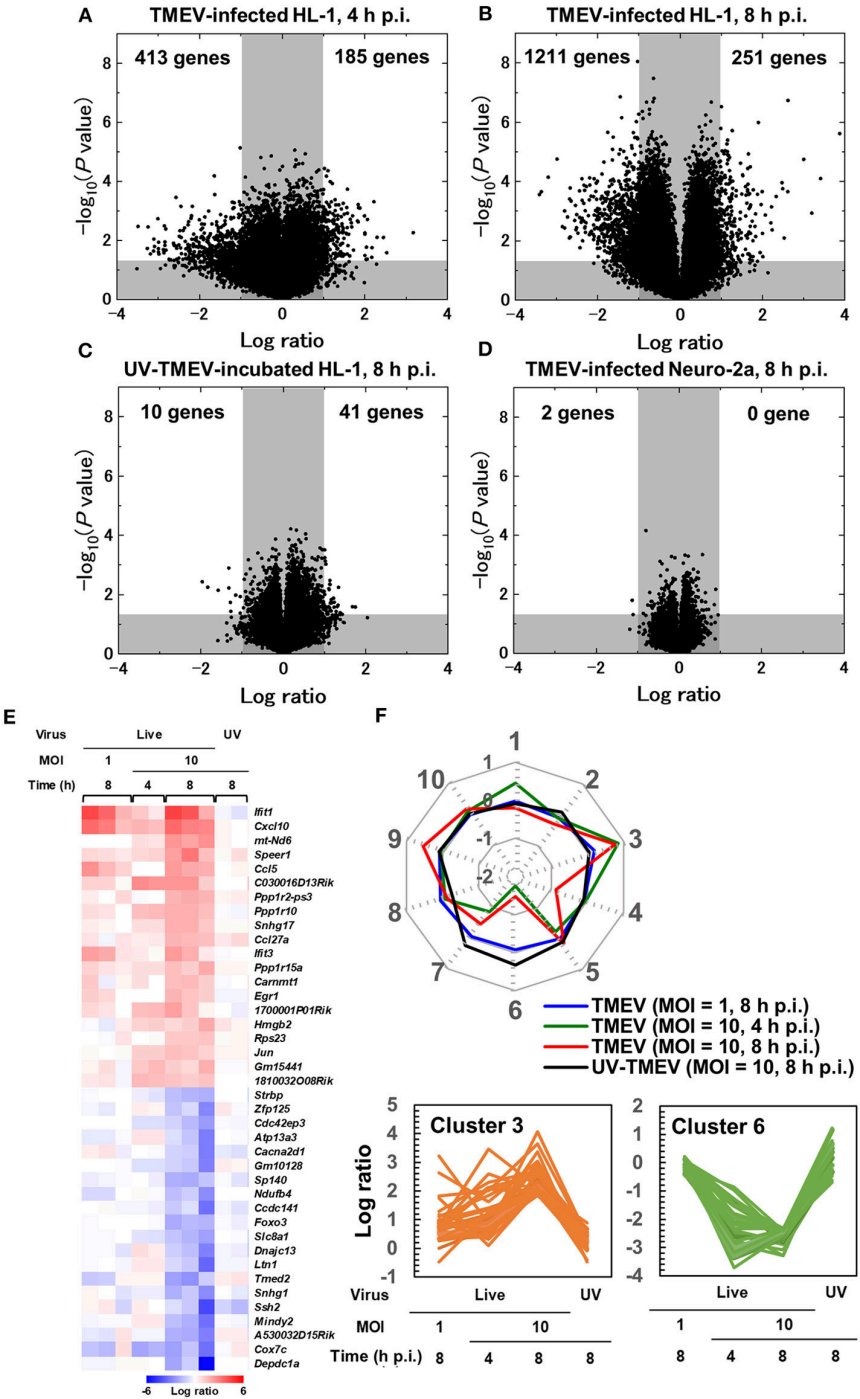
Supervised bioinformatics analysis of transcriptome data from cardiomyocyte HL-1 cells infected with TMEV (33, 42). **(A–D)** Volcano plots of significantly up-regulated (upper right) or down-regulated genes (upper left) in TMEV-infected cells by the OriginPro 8.1 (OriginLab Corporation, Northampton, MA), to assess significance together with log ratio of transcriptome data (Supplementary Materials and Methods) (33). Log ratios of gene expression in the TMEV-infected cell culture compared with mock-infected cell culture were used as the x-axis and the logarithms of *P* values to base 10 were used as the y-axis. **(A)** TMEV-infected HL-1 cells at MOI = 10 at 4 h p.i. **(B)** TMEV-infected HL-1 cells at MOI = 10 at 8 h p.i. **(C)** HL-1 cells incubated with ultraviolet (UV)-irradiated TMEV for 8 h. **(D)** TMEV-infected Neuro-2a cells s at MOI = 10 at 8 h p.i. **(E)** Heat map of 20 up- or down-regulated genes in TMEV-infected HL-1 cells at MOI = 10 at 8 h p.i. by R version 3.4.3 and the R packages “gplots” and “genefilter.” Red, blue, and white indicate up-regulation, down-regulation, and no change, respectively. Interferon-inducible genes (*Ifit1* and *Ifit3*) and chemokines (*Cxcl10* and *Ccl5*) were significantly up-regulated. While TMEV-infected HL-1 cells at MOI = 10 for 4 h or at MOI = 1 for 8 h showed a similar expression pattern in several genes, HL-1 cells incubated with UV-TMEV for 8 h did not up- or downregulated these genes. **(F)** Radar chart based on the values of cluster centers from *k*-means clustering. The number at each vertex is the cluster number (clusters 1 to 10), whereas the numbers along the axis (−2 to 1) are log ratios compared with mock-infected controls. Up-regulated genes in TMEV-infected HL-1 cells 4 and 8 h p.i., including *Cxcl10* and *Ifit1*, were categorized mostly in cluster 3, while the downregulated genes only 8 h p.i. or 4 and 8 h p.i. were categorized in cluster 4 or 6, respectively. In UV-TMEV-incubated HL-1 cells, most genes showed no change; the values of most cluster centers were log ratios = 0. List of genes in each cluster was shown in Supplementary Table 3.

To characterize gene expression patterns in cardiomyocytes infected with TMEV, we conducted a supervised analysis using the 2-way comparison of microarray data between TMEV-infected and control mock-infected HL-1 cell culture samples (Supplementary Materials and Methods). We visualized the numbers of up- or downregulated genes of infected HL-1 cells compared with controls, using a volcano plot (Figures 3A–D) (44–46). We identified substantial numbers of genes whose expressions changed 4 h p.i. (185 upregulated and 413 downregulated genes, >2-fold compared with controls), and their numbers were increased 8 h p.i. (251 upregulated and 1,211 downregulated genes).

To compare these gene expression patterns among samples, we generated the heat map for highly up- or downregulated genes (13), using top 20 of up- or downregulated genes of HL-1 samples 8 h p.i. (Figure 3E). At 8 h p.i., TMEV infection upregulated genes associated with innate immunity: IFN-induced genes, including *Ifit1*, and *Cxcl10* and *Ccl5*. TMEV-infected HL-1 samples 4 h p.i. showed a similar gene expression pattern to that of 8 h p.i. We categorized the genes up- or downregulated in TMEV-infected HL-1 cells, using the Database for Annotation, Visualization, and Integrated Discovery (DAVID) v6.8 (Laboratory of Human Retrovirology and Immunoinformatics, Leidos Biomedical Research, Inc., Frederick, MD). Among the upregulated genes, DAVID identified 18 pathways whose *P* values were <0.05 (Supplementary Table 1), including “chemokine-mediated signaling pathway,” “cellular response to IFN-α and IFN-β,” and “positive regulation of T cell migration.” Among the downregulated genes, DAVID identified 28 pathways, including “cell division” and “heart morphogenesis.”

To determine the requirement of live virus for the gene expression changes, we incubated HL-1 cells with ultraviolet (UV)-irradiated (replication inactive) TMEV (UV-TMEV) (47). Following 8 h incubation, UV-TMEV upregulated 41 genes, among which only one gene *Mir690* was identified, while none of the 41 genes significantly upregulated in live TMEV-infected HL-1 cells (Figure 3C; Supplementary Table 2). UV-TMEV also downregulated 10 genes whose immunological functions are unknown, while one gene [slingshot protein phosphatase 2 (*Ssh2*)] among the 10 genes was also downregulated in live TMEV-infected HL-1 cells. To identify cell-type specific gene expression, we conducted microarray analyses using TMEV- and mock-infected Neuro-2a cells (43). In Neuro-2a cells, TMEV infection did not upregulate any genes significantly, while two genes with unknown functions were downregulated (Figure 3D; Supplementary Table 2). No innate immunity-related genes were induced in HL-1 cells incubated with UV-TMEV or TMEV-infected Neuro-2a cells (Figure 3E; Supplementary Table 2). Thus, induction of innate immunity-related genes by TMEV requires live virus and is cell-type specific.

To identify sets of genes whose expression patterns were unique under the experimental conditions, we conducted *k*-means clustering (Figure 3F) (33). Among 10 clusters, three clusters (clusters 3, 4, and 6) showed differentially expressed patterns, which were visualized by radar chart showing the different expression patterns of cluster centers in each cluster. Most upregulated genes in TMEV-infected HL-1 cells 4 and 8 h p.i., including *Ifit1* and *Cxcl10*, were categorized in clusters 3, while the downregulated genes only 8 h p.i. or 4 and 8 h p.i. were categorized in cluster 4 or 6, respectively. Lists of genes in each cluster were shown in Supplementary Table 3.

### PCA of Microarray Data Separates Between the TMEV-Infected HL-1 and Control Groups

**Figure 4:**
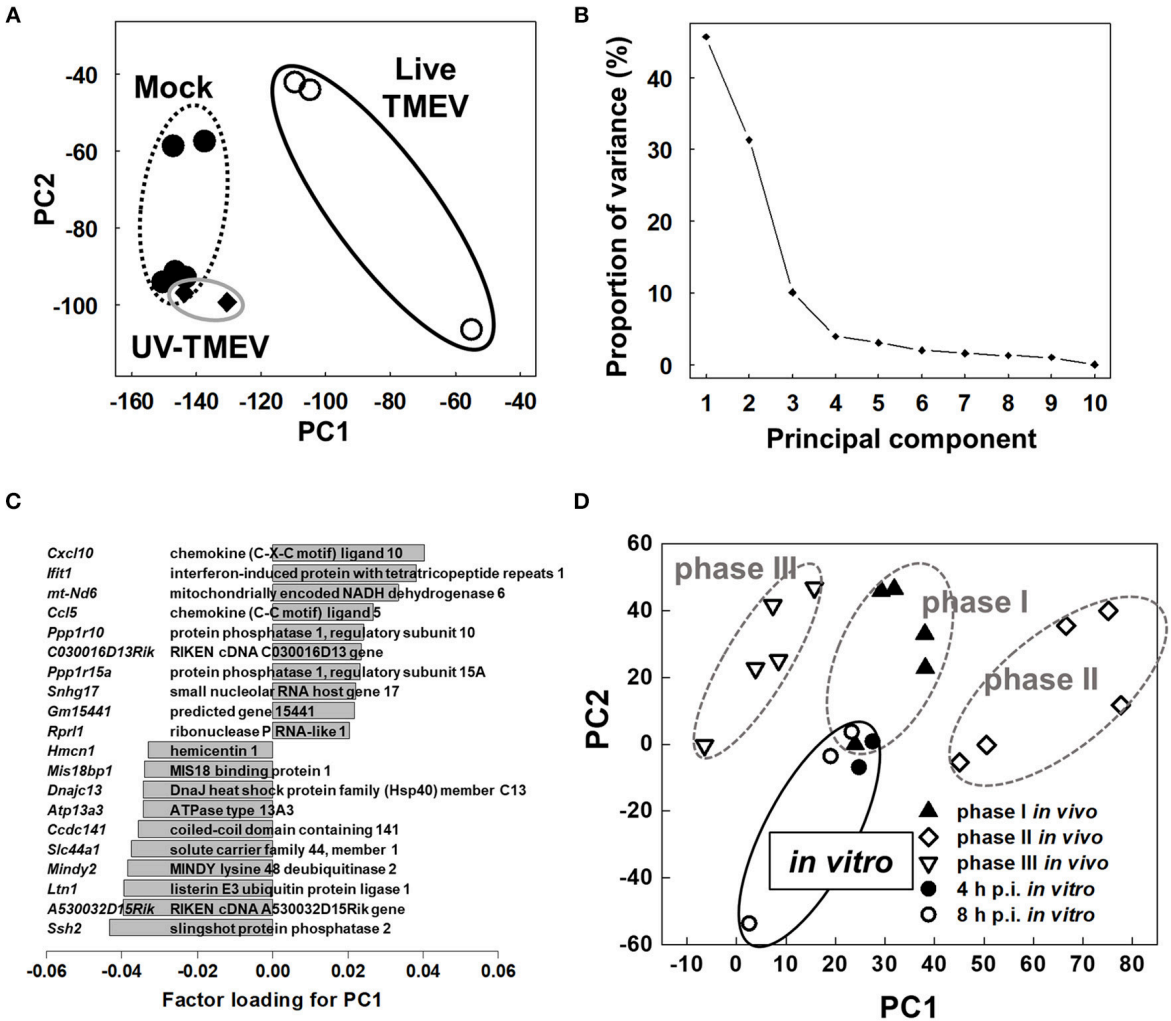
Unsupervised principal component analysis (PCA) of transcriptome data of mock-infected, TMEV-infected, and UV-TMEV-incubated HL-1 cells (33, 42). **(A)** PCA separated samples into two groups: TMEV-infected samples vs. uninfected samples (mock infection and UV-TMEV), where principal component (PC) 1 reflected live virus infection. **(B)** The proportion of variance showed that PC1 explained 46% of the variance among the samples. **(C)** Factor loading for PC1 showed that chemokines (*Cxcl10* and *Ccl5*) and interferon-inducible genes (*Ifit1*) were correlated with PC1 positively, while several genes including slingshot protein phosphatase 2 (Ssh2) and listerin E3 ubiquitin protein ligase 1 (*Ltn1*) were correlated with PC1 negatively. **(D)** PCA of transcriptome data of TMEV-infected HL-1 cells 4 and 8 h p.i. and heart samples from phases I (4 days p.i.), II (7 days p.i.), and III (60 days p.i.) in TMEV-induced myocarditis *in vivo*. PCA showed that phase I samples and *in vitro* samples had similar PC1 values, compared with phases II and III samples. PCA was conducted using R version 3.4.3 (13). Microarray data were converted into tab-delimited text format and calculated using an R program “prcomp”.

To compare overall gene expression patterns among samples, we conducted unsupervised PCA by entering microarray data from each sample without labeling of grouping (33, 42). In PCA, each principal component (PC) is determined automatically, and PC values for each sample data are plotted, for example, PC1 as the x-axis and PC2 as the y-axis. When the data of all HL-1 samples from mock-infection, TMEV-infection, and UV-TMEV incubation were entered, we found that the samples were separated into two distinct populations: live TMEV-infected samples vs. uninfected samples (mock-infection and UV-TMEV) (Figure 4A). According to the proportion of variance, PC1 explained 46% of the variation among samples (Figure 4B). Factor loading for PC1 showed that innate immunity-related genes, including *Cxcl10, Ccl5*, and *Ifit1*, contributed to PC1 positively, while a group of genes, including *Ssh2* (48), listerin E3 ubiquitin protein ligase 1 (*Ltn1*), and MINDY lysine 48 deubiquienase 2 (*Mindy2*) (49), contributed negatively (Figure 4C). Thus, both supervised and unsupervised analyses suggested that innate-immunity-related genes, including *Cxcl10, Ccl5*, and *Ifit1*, could be biomarkers for the differences between the TMEV-infected and control groups *in vitro*.

The gene expression changes in TMEV-infected HL-1 cells appeared to be similar to those found in the heart during phase I of *in vivo* TMEV infection. Thus, we conducted PCA by entering microarray data from TMEV-infected HL-1 cells and those from heart samples of all three phases in TMEV-infection *in vivo* (Figure 4D) (33, 42) to see whether the overall gene expression pattern of TMEV-infected HL-1 cells could be similar to those of TMEV-induced myocarditis *in vivo*. PCA clearly separated *in vivo* samples from three phases into three distinct groups by PC1 values; the PC1 values reflected distinct pathophysiology of three phases of myocarditis. Here, the PC1 values of *in vitro* TMEV-infected HL-1 cells 4 and 8 h p.i. were similar to that of heart samples of phase I in TMEV-induced myocarditis. On the other hand, PC2 values of *in vitro* TMEV-infected HL-1 cells were lower than those of *in vivo* samples. Thus, PC2 values could reflect the differences between *in vitro* and *in vivo* conditions, rather than phase-specific pathophysiology.

## Cytokines and Lymphatics in TMEV Infection

### Cytokines and Lymphatics in TMEV-Induced Myocarditis

Although several cytokines have been shown to influence lymphangiogenesis, the pro-lymphangiogenic cytokine, vascular endothelial growth factor (VEGF)-C or D (50), binds to VEGF receptor (VEGFR) 3 on lymphatic vessel endothelial cells to induce lymphangiogenesis during inflammation (“inflammation-associated lymphangiogenesis,” IAL) (35), where macrophage-secreted VEGF induces sprouting of lymphatic vessels at the preexisting lymphatic vessels (51). The VEGF-A/VEGFR2, which is typically associated with angiogenesis (52), also induces lymphangiogenesis in a context-dependent manner, such as corneal lymphangiogenesis (50).

Cardiac lymphatic networks exist in all three layers of the heart, forming subendocardial, myocardial, and subepicardial plexuses, while these lymphatics share anatomical and physiological characteristics with those in other organs (53). Disturbed cardiac lymphatic drainage can contribute to many forms of cardiac pathology, such as dilated cardiomyopathy and heart failure. Myocarditis provokes myocardial edema and inflammatory infiltration of lymphocytes and macrophages; both can drive underlying lymphatic pumping disturbances. Lymphatic contraction is often impaired by inflammatory mediators, including cytokines, prostaglandins (PGs), and nitric oxide (54); inflammatory mediators produced during myocarditis could depress lymphatic pumping and drainage. In TMEV-induced myocarditis, we previously showed that pro-inflammatory cytokine interleukin (IL)-1β and tumor necrosis factor (TNF)-α upregulation was associated with myocarditis *in vivo* without induction of lymphatic markers, including lymphatic vessel endothelial hyaluronan receptor (LYVE)-1, or VEGFR3 (55). In addition, IL-1β reduced contractility of cardiac lymphatic muscle cells via cyclooxygenase (COX)-2/PGE_2_ signaling with synergistic cooperation by TNF-α *in vitro*. These results suggest that a loss of cardiac lymphatic tonic contractility induced by IL-1β could exacerbate myocardial edema, leading to accumulation of inflammatory cytokines/chemokines and immune cells within the heart, while this may prevent viral spread to the systemic circulation.

### Lymphatics and Virus Infection in the CNS

The CNS has been regarded as an immunologically privileged site due to several characteristics that isolate it from systemic immune responses under physiological conditions: lack of MHC molecules on most resident cells, the presence of the blood-brain barrier (BBB) with low adhesion molecule expression on blood vessels, and no conventional lymphatic system (56). Recently, meningeal lymphatic vessels have been identified in the CNS that may be used for clearance of not only soluble molecules (57) but also immune cells (58) from the CNS and drainage to the deep cervical lymph node. Although there have been many experimental reports showing the transport of soluble molecules, the cellular transport from the CNS to cervical lymph nodes is still controversial. For example, even highly malignant cancer cells in the CNS do not metastasize to any peripheral lymph nodes; cellular transport using the lymphatics from the CNS seems to be regulated with unknown mechanisms. Although the soluble antigens transported from the CNS to cervical lymph nodes can be used for antigen presentation, it is unclear whether this pathway is a major priming site for presentation of CNS antigens since cervical lymph node swelling is not seen in CNS microbial infections or CNS inflammatory diseases.

Using experimental intravenous injection of simian immunodeficiency virus (SIV) in rhesus macaques, Dave et al. (59) demonstrated the presence of SIV in the CNS and cervical lymph nodes with lower levels of virus in plasma, suggesting SIV spread from the CNS to draining cervical lymph nodes. Although the exit of SIV from the CNS via lymphatic vessels should be confirmed by future studies, including the comparison of viral genotypes between the CNS and lymph nodes, this study showed the possibility that lymphatics might be used for virus clearance and/or exit from the CNS to the periphery.

### Lymphocyte Entry/Exit and Lymphatics in CNS TMEV Infection

**Figure 5:**
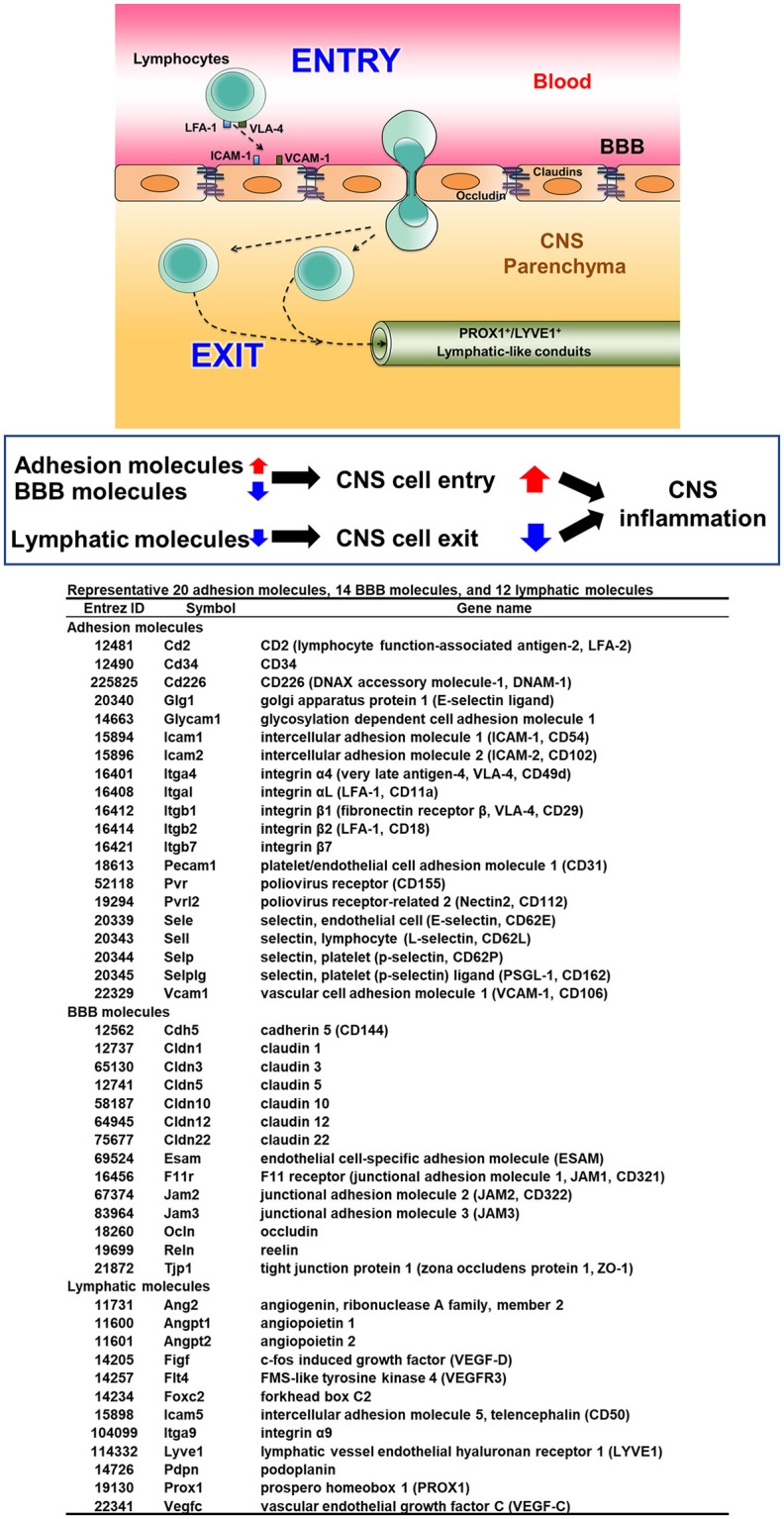
Three components of lymphocyte entry into and exit from the CNS (61, 62). To initiate inflammation in the CNS, lymphocytes interact with endothelial cells of blood vessels via up-regulated adhesion molecules, particularly very late antigen (VLA)-4 and lymphocyte function-associated antigen (LFA)-1 on lymphocytes with vascular cell adhesion molecule (VCAM)-1 and intercellular adhesion molecule (ICAM)-1 on endothelia, respectively. Downregulation of molecules composed of the blood-brain barrier (BBB) also help in lymphocyte entry into the CNS parenchyma. While the precise mechanism of lymphocyte exit from the CNS is unknown, one hypothesis is the presence of vessels similar to peripheral lymphatic vessels, whose markers include prospero homeobox (PROX) 1 and lymphatic vessel endothelial hyaluronan receptor (LYVE) 1, may help in lymphocyte exit from the CNS to deep cervical lymph nodes. Increased lymphocyte entry together with decreased lymphocyte exit could lead to enhancement of CNS inflammation.

In MS and its animal models, the presence of immune cell infiltrates, particularly lymphocytes, in the CNS has been correlated with disease activity and neuropathology. Lymphocyte entry into the CNS is accompanied by upregulation of adhesion molecules on lymphocytes and blood vessels as well as a breakdown of the BBB (60) (Figure 5). Among the adhesion molecules, the interactions between very late antigen (VLA)-4 (CD49d/CD29) and vascular cell adhesion molecule (VCAM)-1 (CD106) (63) as well as leukocyte function-associated antigen (LFA)-1 (CD11a/CD18) and intercellular adhesion molecule (ICAM)-1 (CD54) (64) have been shown to play a key role for lymphocyte extravasation into the CNS parenchyma (63). The BBB is composed of tight junctions of endothelial cells, the basement membrane, and astrocyte foot processes. Downregulation of tight junction proteins, including occludin and claudin, has been associated with BBB breakdown and disease activities in MS and its animal models (62). On the other hand, the pathophysiology of lymphocyte exit from the CNS is unclear, although newly identified CNS lymphatic vessels (58) might contribute to clearance of lymphocytes (and microbes) from the CNS, in theory.

**Figure 6:**
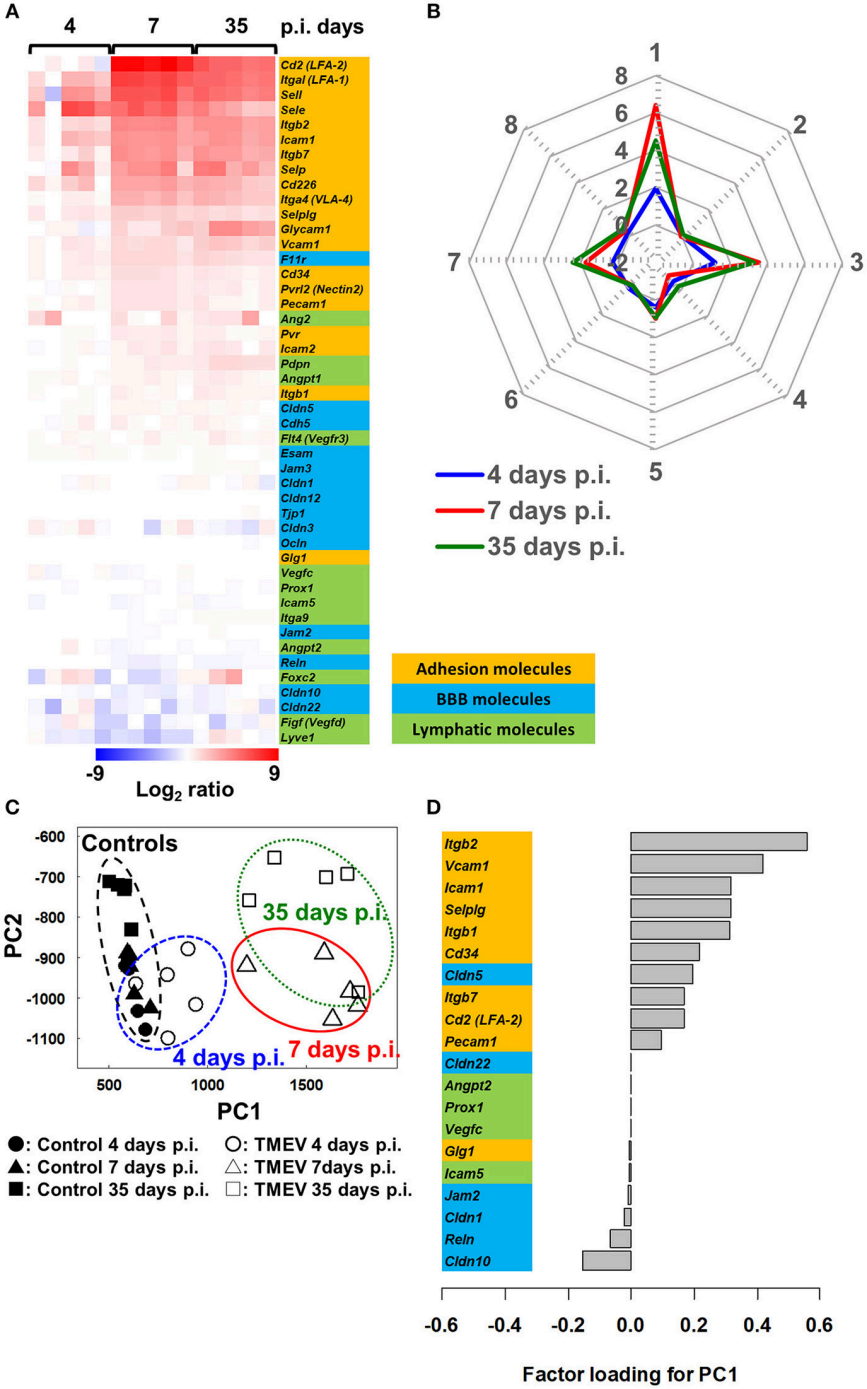
Bioinformatics analyses of gene expression of three components associated with spinal cord inflammation in TMEV infection, 4 (prior to cell infiltration), 7 (acute polioencephalomyelitis) and 35 (TMEV-IDD) days p.i. (61). **(A)** We drew a heat map, using mRNA data of 20 adhesion molecules, 14 BBB molecules, and 12 lymphatic molecules listed in Figure 5 (total 46 genes). Most adhesion molecule genes were upregulated 7 and 35 days p.i., while only a few adhesion molecules were upregulated 4 days p.i. BBB and lymphatic molecules showed no change or slight downregulation. **(B)** Radar chart based on the values of cluster centers from *k*-means clustering (Supplementary Table 4). The number of each vertex is the cluster number (clusters 1 to 8), whereas the number along the axis (−2 to 8) are log ratios compared with mock-infected controls. Radar chart showed that the expression patterns of sets of genes were similar between days 7 and 35 p.i. Upregulated genes were categorized mostly in clusters 1, 3, and 7. **(C)** PCA of the 46 genes listed in Figure 5 separated controls/day 4 p.i. samples vs. days 7 and 35 p.i. samples based on PC1 values (proportion of variance was 85.9%), which reflect CNS cell infiltration. **(D)** Factor loading for PC1 showed that upregulation of adhesion molecules was associated with CNS inflammation, while downregulation of BBB and lymphatic molecules may play a minor role.

In TMEV infection, we determined the extent of which expressions of the adhesion molecules, BBB and lymphatic molecules could be associated with CNS disease activity (53, 61). Using the RNA sequencing transcriptome data from the spinal cord of TMEV-infected mice harvested 4, 7, and 35 days p.i. (Supplementary Materials and Methods), we compared mRNA levels of representative 20 lymphocyte and vascular adhesion molecules, 14 BBB molecules, and 12 lymphatic molecules among samples (Figure 5). Both 7 and 35 days p.i., heat map showed that most adhesion molecules were upregulated, while lymphatic and BBB molecules showed no change or slight downregulation (Figure 6A). Since samples 7 days p.i. contain gray matter inflammatory lesions due to acute polioencephalomyelitis and those 35 days p.i. contain inflammatory demyelination in the white matter, we expected substantial difference in gene expression patterns between the two sample groups. Unexpectedly, however, the levels of most adhesion molecules 7 days p.i. were similar or slightly higher, compared with 35 days p.i. Only glycosylation-dependent cell adhesion molecule (GLYCAM) 1 was significantly upregulated from 7 to 35 days p.i. (65). Thus, GLYCAM1 may have a role in chronic demyelination. Most genes 4 days p.i. showed no or few changes, which is consistent with the histological finding that immune cell infiltrates become obvious 5 days p.i. in CNS TMEV infection. In radar chart that visualized gene expression patterns by *k*-means clustering, clusters 1, 3, and 7 were composed of highly upregulated genes 7 and 35 days p.i. (cluster 1, LFA-1 and 2, E- and L-selectin; cluster 3, ICAM-1, and other molecules; and cluster 7, VLA-4, VCAM-1, and GLYCAM1) (Figure 6B; Supplementary Table 4). Cluster 4 was composed of downregulated genes 4, 7, and 35 days p.i., including VEGF-C, LYVE1, and claudin 22.

We also conducted PCA using the same 46 gene expression data and found that expression patterns of molecules associated with CNS lymphocyte entry and exit could distinguish samples without CNS cell infiltration (control and 4 day p.i. samples) vs. with CNS cell infiltration (7 and 35 days p.i. samples) by PC1 values (Figure 6C). Factor loading for PC1 showed that upregulation of adhesion molecules (66) was correlated with PC1 values that reflect CNS inflammation 7 and 35 days p.i. (Figure 6D). Downregulation of several BBB molecules, including claudin 10 (67) and reelin, was weakly correlated with PC1 values. Downregulation of BBB may play a minor role in CNS inflammation induced with TMEV, although downregulation of BBB molecules has been reported not only in MS and autoimmune model for MS but also in another experimental CNS viral model induced with mouse hepatitis virus (68).

Inflammation has been reported to induce lymphangiogenesis in several organs and tissues. Following intracerebral TMEV infection in the CNS, however, most lymphatic markers were not upregulated at any time points, although the constitutive expression in control uninfected CNS tissues supports the presence of lymphatic-like structure in the CNS. This is consistent with our previous findings on the protein levels of lymphatic biomarkers, in which there was no increase in lymphatic markers, LYVE1 or prospero homeobox protein (PROX)1 in the CNS of TMEV-IDD (39). Most lymphatic molecules were actually downregulated slightly on 7 and 35 days p.i., while factor loading for PC1 showed that downregulation of lymphatic molecules was weakly correlated with PC1 values. This suggests that dysfunction of lymphatic-like structure might delay exit of inflammatory cytokines/chemokines and/or cells from the CNS, enhancing inflammation, only to some extent. On 14 days p.i. when inflammation had subsided in the CNS, the levels of most lymphatic molecules of the TMEV-infected spinal cord were similar to those of uninfected control spinal cord (data not shown); this may reflect that recovery of lymphatic flow from the CNS contributes to exit of inflammatory cytokines/chemokines and/or cells from the CNS around 2 weeks p.i.

In TMEV-IDD, the balance between lymphocyte entry and exit could play a key role in inflammation in the CNS; upregulation of adhesion molecules rather than downregulation of BBB molecules could contribute to lymphocyte entry, while downregulation of lymphatic molecules may play a minor role in prolonged inflammation. In theory, dysfunction of the lymphatics results in the persistence of lymphocytes and cytokines/chemokines in the CNS (69). This would lead to chronic inflammation and immune-mediated demyelination by immunopathology, whereas such lymphostasis might confine TMEV to the CNS, limiting systemic viral spreading. Here, virus-specific lymphocytes among chronic cellular infiltrates in the CNS may also minimize virus replication in the CNS.

In summary, in TMEV infection, innate immune cytokines may play distinctive and diverse roles in lymphatic networks during inflammatory disease depending on the organs, which contribute to the levels of inflammation and to virus persistence (Table 1). Although TMEV can infect major cell types of the CNS (neurons) and the heart (cardiomyocytes), only infected cardiomyocytes expressed innate immunity-related molecules. In addition, lymphatic vessels in infected organs may also be differentially affected between the CNS and the heart. In the heart of TMEV-induced acute myocarditis, IL-1β with TNF-α could functionally alter lymphatics, while downregulation of lymphatic molecules might contribute to persistent virus infection and inflammation in the CNS of TMEV-IDD. These potential factors may contribute to organ-specific viral immunopathology in TMEV infection.

**Table 1:** Potential factors contributing to TMEV-induced organ-specific pathology.

	**CNS**	**Heart**
Infection of major cell type *in vitro*	+ (Neuro-2a)	+ (HL-1)
Innate immune response by major cell type *in vitro*	–	+
Infection *in vivo*	+	+
Lymphatics	Lymphatic molecule downregulation?	Cytokine-induced functional suppression

## Ethics Statement

This study was carried out in accordance with the recommendations of the criteria outlined by the National Institutes of Health (NIH). The protocol was approved by the Institutional Animal Care and Use Committee of LSUHSC-S and Kindai University.

## Author Contributions

IT and SO for substantial contributions to the conception or design of the work. SO, EK, FS for the acquisition of data. SO, UC, and MT for analysis of data. IT, AM, MA-K, and JA for interpretation of data for the work. IT, SO, NM, JY, and JA for drafting the work or revising it critically for important intellectual content.

### Conflict of interest statement

The authors declare that the research was conducted in the absence of any commercial or financial relationships that could be construed as a potential conflict of interest.
