# Supplementary material for: Theiler's Virus-Mediated Immunopathology in the CNS and Heart: Roles of Organ-Specific Cytokine and Lymphatic Responses
Source: Front Immunol. 2018 Dec 10;9:2870. doi: 10.3389/fimmu.2018.02870 (PMC6295469; doi:10.3389/fimmu.2018.02870)
Supplement: Supplementary file 1 [file Data_Sheet_1.zip › Data Sheet 1/Supplementary Materials and Methods.DOCX]

***Supplementary Data***

**Theiler’s Virus-Mediated Immunopathology in the CNS and Heart:**

**Roles of Organ-Specific Cytokine and Lymphatic Responses**

Seiichi Omura, Ph.D., Eiichiro Kawai, M.D., Ph.D., Fumitaka Sato, Ph.D., Nicholas E. Martinez, Ph.D., MBA, Alireza Minagar, M.D. , Mahmoud Al-Kofahi, Ph.D. , J. Winny Yun, Urska Cvek, Sc.D., MBA, Marjan Trutschl, Sc.D., J. Steven Alexander, Ph.D. , Ikuo Tsunoda, M.D., Ph.D.

***Correspondence:** Ikuo Tsunoda, M.D., Ph.D.: itsunoda@med.kindai.ac.jp

**MATERIALS AND METHODS**

**Animal experiments**

Female 5-week-old SJL/J mice (Jackson Laboratory, Bar Harbor, ME) were infected intracerebrally with 2 × 10^5^ plaque forming units (PFU) of the Daniels (DA) strain of TMEV. Animals were maintained under specific pathogen-free conditions in our animal care facility at Louisiana State University Health Sciences Center-Shreveport (LSUHSC-S), LA, USA, or Kindai University Faculty of Medicine, Osaka, Japan. All experimental procedures involving the use of animals were conducted according to the criteria outlined by the National Institutes of Health (NIH) and were approved by the Institutional Animal Care and Use Committee of LSUHSC-S and Kindai University.

**RNA preparation**

TMEV-infected and uninfected HL-1 cells were homogenized with a QIAshredder (Qiagen, Valencia, CA). The mouse spinal cords were homogenized in TRI-Reagent (Molecular Research Center, Cincinnati, OH). Total RNA was extracted with an RNeasy Mini Kit (Qiagen) according to the manufacturer’s instructions. DNase treatment was performed during RNA isolation with an RNase-Free DNase Set (Qiagen). All samples were purified to an absorbance ratio (A260/A280) between 1.9 and 2.1.

**Transcriptome analysis**

For microarray analysis, we reverse-transcribed 100 ng of total RNA to first-strand cDNA using a Poly-A RNA Control (Affymetrix, Santa Clara, CA) and Ambion WT Expression Kit (Life Technologies Corporation, Carlsbad, CA). The double-stranded cDNA was generated from single-stranded cDNA and transcribed *in vitro* into cRNA. The resulting cRNA was purified and 10 μg of purified cRNA was reverse-transcribed again into 2^nd^-cycle cDNA. 2^nd^-cycle cDNA was fragmented and labeled with terminal deoxynucleotidyl transferase using Affymetrix proprietary DNA Labeling Reagent (Affymetrix). Labeled cDNA was hybridized to a GeneChip Mouse 1.0ST Array (Affymetrix). Hybridized arrays were washed and stained using the GeneChip Hybridization Wash and Stain Kit (Affymetrix) and Fluidics station 450 (Affymetrix), and scanned using Affymetrix GeneChip Scanner 3000 (Affymetrix). Data were visualized and quantified by Affymetrix GeneChip Command Console (AGCC), and normalized by Robust Multi-array Average (RMA) using Expression Console (Affymetrix).

For RNA sequencing (RNA-seq), we synthesized mRNA library from 100 ng of total RNA, using a TruSeq Stranded Total RNA LT Sample Prep kit - Set A with Ribo-Zero Gold (Illumina, Inc, San Diego, CA). The mRNA libraries were validated, using the Agilent 4200 TapeStation (Agilent Technologies, Santa Clara, CA), and were sequenced on the Illumina NextSeq 500 instrument, according to manufacturer’s instruction. The sequencing was performed, using Single Read configuration. The raw sequence data were aligned to reference genome, Mus musculus GRCm38.75, downloaded from ENSEMBLE, using a software “Spliced Transcripts Alignment to a Reference” (STAR) ([Dobin et al., 2013](#_ENREF_3)). The read count data were normalized with two methods: 1) read counts per kilobase (RPK) and 2) differentially expressed gene elimination strategy (DEGES), using “R” and the package “tag count comparison (TCC)” ([Sun et al., 2013](#_ENREF_6)).

Both microarray and RNA-seq data were analyzed using the Database for Annotation, Visualization and Integrated Discovery (DAVID) v6.8 [Laboratory of Human Retrovirology and Immunoinformatics (LHRI); https://david.ncifcrf.gov/home.jsp] ([Huang da et al., 2009](#_ENREF_4)), NetAffx database (Affymetrix; http://www.affymetrix.com/index.affx), and Mouse Genome Informatics (The Jackson Laboratory, Bar Harbor, ME; http://www.informatics.jax.org/). Transcriptome data have been deposited into the Gene Expression Omnibus at the National Center for Biotechnology Information (Bethesda, MD, USA; accession no. GSE120042, https://www.ncbi.nlm.nih.gov/geo/query/acc.cgi?acc=GSE120042).

**Bioinformatics analyses**

**Volcano plot:** We drew a volcano plot, using the OriginPro 2018 (OriginLab Corporation, Northampton, MA), to visualize significance and fold changes of transcriptome data.

**Heat map:** We drew heat maps to determine the expression patterns of top 20 up- or down-regulated genes in TMEV-infected HL-1 cells and spinal cords from TMEV-infected mice, and compared the expression levels between TMEV infection versus control groups, using R version 3.5.1 and the programs ‘gplots’ and ‘genefilter’ .

***K*-means clustering:** We conducted *k*-means clustering, using an R package ‘cclust’ ([Chaitanya et al., 2013](#_ENREF_1); [Omura et al., 2014](#_ENREF_5)). We used Davies-Bouldin index to determine the optimum number of clusters ([Davies and Bouldin, 1979](#_ENREF_2)).

**Principal component analysis (PCA):** PCA was conducted, using an R program ‘prcomp’, as we described previously ([Chaitanya et al., 2013](#_ENREF_1); [Omura et al., 2014](#_ENREF_5)). The proportion of variance was also calculated to determine the percentage of variance explained by each principal component (PC), while factor loading for PC1 was used to rank a set of genes contributing to PC1 values.

**REFERENCES**

Chaitanya, G.V., Omura, S., Sato, F., Martinez, N.E., Minagar, A., Ramanathan, M., et al. (2013). Inflammation induces neuro-lymphatic protein expression in multiple sclerosis brain neurovasculature. *J Neuroinflammation* 10**,** 125. doi: 10.1186/1742-2094-10-125.

Davies, D.L., and Bouldin, D.W. (1979). A cluster separation measure. *IEEE Trans Pattern Anal Mach Intell* 1(2)**,** 224-227.

Dobin, A., Davis, C.A., Schlesinger, F., Drenkow, J., Zaleski, C., Jha, S., et al. (2013). STAR: ultrafast universal RNA-seq aligner. *Bioinformatics* 29(1)**,** 15-21. doi: 10.1093/bioinformatics/bts635.

Huang da, W., Sherman, B.T., and Lempicki, R.A. (2009). Systematic and integrative analysis of large gene lists using DAVID bioinformatics resources. *Nat Protoc* 4(1)**,** 44-57. doi: 10.1038/nprot.2008.211.

Omura, S., Kawai, E., Sato, F., Martinez, N.E., Chaitanya, G.V., Rollyson, P.A., et al. (2014). Bioinformatics multivariate analysis determined a set of phase-specific biomarker candidates in a novel mouse model for viral myocarditis. *Circ Cardiovasc Genet* 7(4)**,** 444-454. doi: 10.1161/CIRCGENETICS.114.000505.

Sun, J., Nishiyama, T., Shimizu, K., and Kadota, K. (2013). TCC: an R package for comparing tag count data with robust normalization strategies. *BMC Bioinformatics* 14**,** 219. doi: 10.1186/1471-2105-14-219.
